# The Metagenomic Analysis of Viral Diversity in Colorado Potato Beetle Public NGS Data

**DOI:** 10.3390/v15020395

**Published:** 2023-01-30

**Authors:** Maria Starchevskaya, Ekaterina Kamanova, Yuri Vyatkin, Tatyana Tregubchak, Tatyana Bauer, Sergei Bodnev, Ulyana Rotskaya, Olga Polenogova, Vadim Kryukov, Denis Antonets

**Affiliations:** 1 State Research Center of Virology and Biotechnology “Vector”, Rospotrebnadzor, 630559 Koltsovo, Russia; 2 Novel Software Systems LLC, Akademika Lavrentiev ave. 6, 630090 Novosibirsk, Russia; 3 Institute of Systematics and Ecology of Animals SB RAS, Frunze str. 11, 630091 Novosibirsk, Russia; 4 Department of Natural Sciences, Novosibirsk State University, Pirogova str. 2, 630090 Novosibirsk, Russia; 5 MSU Institute for Artificial Intelligence, Lomonosov ave. 27, 119192 Moscow, Russia

**Keywords:** *Leptinotarsa decemlineata*, insect viruses, metagenomic analysis, NGS, endogenous viral elements

## Abstract

The Colorado potato beetle (CPB) is one of the most serious insect pests due to its high ecological plasticity and ability to rapidly develop resistance to insecticides. The use of biological insecticides based on viruses is a promising approach to control insect pests, but the information on viruses which infect leaf feeding beetles is scarce. We performed a metagenomic analysis of 297 CPB genomic and transcriptomic samples from the public National Center for Biotechnology Information Sequence Read Archive (NCBI SRA) database. The reads that were not aligned to the reference genome were assembled with metaSPAdes, and 13314 selected contigs were analyzed with BLAST tools. The contigs and non-aligned reads were also analyzed with Kraken2 software. A total of 3137 virus-positive contigs were attributed to different viruses belonging to 6 types, 17 orders, and 32 families, matching over 97 viral species. The annotated sequences can be divided into several groups: those that are homologous to genetic sequences of insect viruses (*Adintoviridae*, *Ascoviridae*, *Baculoviridae*, *Dicistroviridae*, *Chuviridae*, *Hytrosaviridae*, *Iflaviridae*, *Iridoviridae*, *Nimaviridae*, *Nudiviridae*, *Phasmaviridae*, *Picornaviridae*, *Polydnaviriformidae*, *Xinmoviridae* etc.), plant viruses (*Betaflexiviridae*, *Bromoviridae*, *Kitaviridae*, *Potyviridae*), and endogenous retroviral elements (*Retroviridae*, *Metaviridae*). Additionally, the full-length genomes and near-full length genome sequences of several viruses were assembled. We also found sequences belonging to *Bracoviriform* viruses and, for the first time, experimentally validated the presence of bracoviral genetic fragments in the CPB genome. Our work represents the first attempt to discover the viral genetic material in CPB samples, and we hope that further studies will help to identify new viruses to extend the arsenal of biopesticides against CPB.

## 1. Introduction

The metagenomic approach has proven to be a highly efficient way of detecting viruses in a variety of environments [1,2]. Unlike traditional cultural and diagnostic methods, viral metagenomics uses high-throughput next-generation sequencing, which makes it possible to identify rare and new viruses and to establish ecological links between microorganisms and their natural habitat [3]. Among the various routes of virus transmission, insect transmission is one of the most widespread. Insects are the largest group of animals on the planet and are of significant ecological, agricultural, and medical importance [2]. However, our knowledge of insect viruses is still limited. Establishing an insect virome will provide new insights into the ecology and evolution of viruses and help address public health issues in the fight against arbovirus infections. In addition, pathogenic insect viruses can be used for the biological control of insect pests, and, thus, the identification of new insect viruses can expand the arsenal of potential biocontrol agents [2]. 

The Colorado potato beetle is one of the most widespread and destructive potato pests in the world. Its ability to adapt to various Solanaceae plants, high ecological plasticity, flexible life-cycle, and rapid development of insecticide resistance have led to its global expansion [4]. During its lifetime, the Colorado potato beetle female can lay up to 800 eggs and produce up to three generations per year, depending on climatic conditions [5]. Unless insecticides are used, the Colorado potato beetle can cause potato crop losses of 40–80% [6]. Although the use of insecticides has led to a sharp decline in the beetle’s populations, its development of resistance to all chemicals registered to date has been observed. Increasing the dosage provides a short-term improvement but it significantly increasing the rate of resistance development [7]. Several research groups have established the reasons for such rapid evolutionary changes when studying the genetic basis of the resistance of the Colorado potato beetle to insecticides and its adaptation to environmental factors [4]. Given that approximately 17% of its genome is composed of mobile genetic elements and that there is a high level of nucleotide diversity in fast-growing populations, the Colorado potato beetle evolves faster than many other beetles [8]. Its high ability to acclimatize is related to specific genes associated with changes in terms of metabolism, growth, and diapause in insects [4]. The mechanisms for the detoxification of secondary plant compounds and, most likely, insecticidal compounds are associated with a wide range of highly variable genes, including genes for carboxylesterase, glutathione-S-transferase, and cytochrome P450, with the Colorado potato beetle’s ability to feed on various Solanaceae plants involving the expansion of genes encoding the digestive enzymes and bitter taste receptors [8]. Colorado potato beetles are expected to develop resistance to all newly introduced insecticides [7]. In addition, the widespread use of chemical pesticides can lead to serious environmental problems because of their nonspecific effects on many other animal species.

Biological control methods are the most environmentally friendly ways to fight various pests of agricultural crops [9,10]. The release of biological control agents to manage pest populations has great potential, but only a small number of natural enemies can be grown at scale. In addition, the development of the natural enemy population is often slower than that of the target insect, usually requiring different conditions for optimal growth [11]. Currently, the existing biological control methods use pathogenic fungi, such as *Beauveria bassiana, Metarhizium* species [12], and bacteria *Bacillus thuringiensis* [13], with no biological control agents based on viruses. The only exceptions are those preparations based on viruses of the family *Baculoviridae,* which are widely used to control insect pests of the order Lepidoptera [14].

Currently, over 20 virus families are known to affect different groups of insects [15]. Most insect virus families cause arbovirus infections and, together with plant viruses, can be transmitted to humans [16]. Nevertheless, many insect viruses are not dangerous to humans. These are used for controlling insect pests, both as model objects and as platforms for developing vector systems [2].

Leaf beetles of the family Chrysomelidae are assumed to be affected by viruses of the families *Baculoviridae* and *Iridoviridae* [17]. The sources in the literature on viruses affecting *L. decemlineata* are extremely scarce. Despite being unquestionably significant for agriculture, the Colorado potato beetle virome is practically unstudied, and our study is the first attempt to detect viral genetic material in the genomic and transcriptomic data of *L. decemlineata* samples (obtained from the NCBI SRA database). Such studies could help to improve our understanding of the ecology of the Colorado potato beetle and may help to identify new viruses to enrich the arsenal of biopesticides and eventually reduce economic losses in agriculture.

## 2. Materials and Methods

### 2.1. Genome and Transcriptome Data of Leptinotarsa Decemlineata Samples

The data on 307 samples of *L. decemlineata* were obtained from the NCBI SRA database, with 297 samples selected for the study, including the genomic data obtained from muscle tissues (PRJNA508767, PRJNA369863, and PRJNA580490) and whole insect tissues (PRJNA171749) as well as the transcriptomic data obtained from antennae tissues (PRJNA280017), heads (PRJNA400685), intestines (PRJNA400685, PRJNA336167), larvae (PRJNA275431 and PRJNA694179), and whole insect tissues (PRJNA438159, PRJNA384383, PRJNA297027, PRJNA464380, PRJNA275662, PRJNA353242, PRJNA646009, and PRJNA553565). The sample sizes of the transcriptomic data varied from 217 Mbp to 20.5 Gbp, with genomic data ranging from 7.1 Gbp to 20.5 Gbp. The genomic samples were sequenced with Illumina HiSeq 2000 (PRJNA508767, PRJNA369863, and PRJNA171749) and Illumina HiSeq 1500 (PRJNA580490). The transcriptomic samples were sequenced with Illumina HiSeq 4000 (PRJNA438159), Illumina HiSeq 2000 (PRJNA280017, PRJNA400685, PRJNA400685, and PRJNA336167), Illumina HiSeq 2500 (PRJNA275431, PRJNA438159, PRJNA384383, PRJNA297027, PRJNA464380, PRJNA275662, PRJNA353242, and PRJNA646009), and Illumina NextSeq 500 (PRJNA553565). The primary data sources are presented in Supplementary Table S1. Project PRJNA171749 [8] was aimed at assembling and annotating the *L. decemlineata* genome, and the data from the PRJNA400685 and PRJNA297027 [18] projects was primarily used to assemble and to annotate the Colorado potato beetle transcriptome.

### 2.2. Databases

1. The NCBI SRA database (https://www.ncbi.nlm.nih.gov/sra, accessed on June 2021).
2. The BigViralDB—our custom database—combined nonredundant viral sequences assembled from four databases: NCBI RefSeq (11,566 viral sequences, June 2021) (https://www.ncbi.nlm.nih.gov/refseq/, accessed on June 2021), Virxicon (327,932 viral sequences, June 2021) [19], Virosaurus (823,421 viral sequences, April 2020) [20], and virus sequences from the NCBI Nucleotide database (1,367,485 viral sequences, June 2021). The database can be provided at request.
3. The NCBI Genome Database (https://www.ncbi.nlm.nih.gov/genome/, accessed on July 2021).
4. The NCBI nt database (https://ftp.ncbi.nlm.nih.gov/blast/db/FASTA/nt.gz, accessed on July 2021).
5. The NCBI nr database (https://ftp.ncbi.nlm.nih.gov/blast/db/FASTA/nr.gz, accessed on July 2021).
6. The NCBI UniVec database (https://www.ncbi.nlm.nih.gov/tools/vecscreen/univec/, accessed on June 2021), a collection of unique vector sequences used in genetic engineering and biotechnology research (3137 sequences, June 2021).

### 2.3. Metagenome Assembly and Sequence Classification

**Figure 1:**
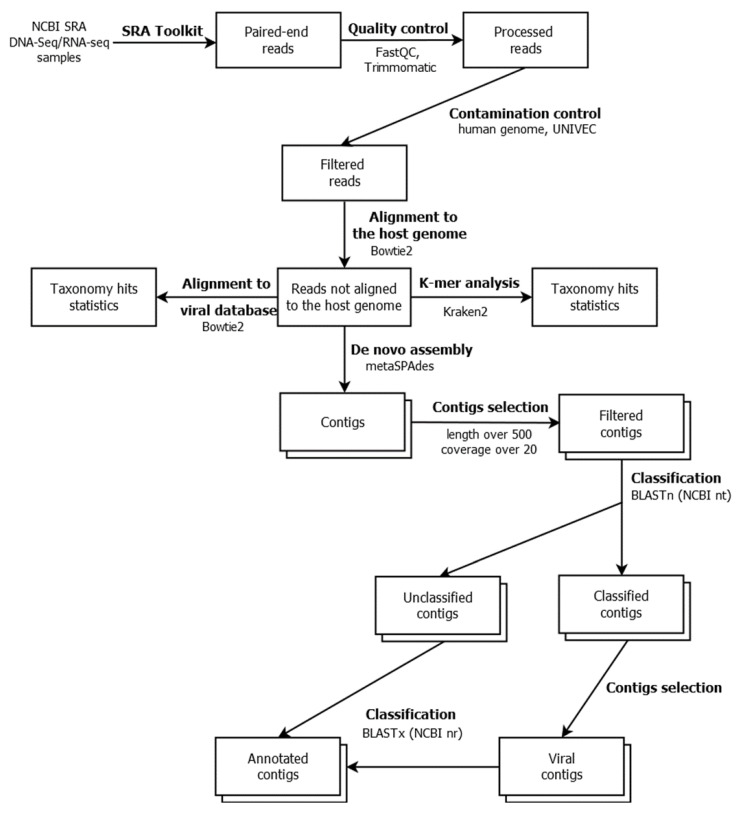
The software pipeline used in this work, with the analysis steps and their connections indicated and the programs used specified. https://github.com/starchevskayamaria17/uncoVir, accessed on December 2022.

We constructed an analytical pipeline to analyze the genomic and transcriptomic data of *Leptinotarsa decemlineata* obtained from the NCBI SRA database (Figure 1). The pipeline, written in Snakemake [21], involves several approaches for classifying genetic sequences and is freely available at: https://github.com/starchevskayamaria17/uncoVir, accessed on December 2022.

In the first step, low-quality and short reads (less than 20 nucleotides in terms of quality and less than 30 nucleotides in terms of length), overrepresented sequences, and adapters were removed using the Trimmomatic tool [22]. For contamination control, the reads aligned to the human genome (GRCh38.p12) using the Bowtie2 tool were deleted [23]. The reads of synthetic origin (identified with BLAST against the UniVec database) were also deleted. The reads aligned to the reference *L. decemlineata* genome (assembly GCA_000500325.2 Ldec_2.0) using the Bowtie2 tool were also excluded from the subsequent analysis.

In the next step, viral diversity was assessed using the k-mer spectrum analysis. The unaligned reads were classified with Kraken2 [24] using our custom compiled viral sequence database BigViralDB. The results were visualized using Pavian [25]. To assess the completeness of particular viral genomes, we also aligned the reads using Bowtie2 to the viral genomic sequences contained in our BigViralDB database.

The reads unaligned on the *L. decemlineata* genome were assembled into contigs using the SPAdes assembler tool [26]. Contigs longer than 500 nucleotides and with coverages of more than 20 were selected and analyzed using BLASTn against the NCBI Nucleotide database. The unclassified contigs and contigs annotated with BLASTn as virus-positive were then analyzed using BLASTx against the NCBI nonredundant protein database. The obtained annotation results were analyzed and visualized using custom scripts written in the Python programming language. The details of the running parameters of the programs were included in the pipelines and are given in the source code in the repository (https://github.com/starchevskayamaria17/uncoVir, accessed on December 2022).

### 2.4. Analysis of Reference Genomes of Selected Coleopterans

The genome sequence of *Leptinotarsa decemlineata* (assembly GCA_000500325.2 Ldec_2.0) used as a reference for alignment was split into 31-nucleotide long k-mers and analyzed using the BLASTn tool against the NCBI Nucleotide database. Similarly, other reference genomes of Coleoptera obtained from the NCBI Genome database were examined: *Diabrotica virgifera* (GCA_003013835.2 Dvir_v2. 0), *Oryzaephilus surinamensis* (GCA_004796505.1 ASM479650v1), *Coccinella septempunctata* (GCA_003568925.1 Csep_MD8_v1), *Harmonia axyridis* (GCA_011033045. 1), *Aleochara bilineata* (GCA_003054995.1 ASM305499v1), *Aethina tumida* (GCA_001937115.1 Atum_1.0), *Oryctes borbonicus* (GCA_902654985. 1), *Nicrophorus vespilloides* (GCA_001412225.1 Nicve_v1.0), *Asbolus verrucosus* (GCA_004193795.1 BDFB_1.0), *Protaetia brevitarsis* (GCA_004143645. 1 ASM414364v1), *Popillia japonica* (GCA_004785975.1 GSC_JBeet_1), *Anoplophora glabripennis* (GCA_000390285.2 Agla_2. 0), *Pogonus chalceus* (GCA_002278615.1 Pchal_1.0), *Agrilus planipennis* (GCA_000699045.2 Apla_2.0), *Sitophilus oryzae* (GCA_002938485.2 Soryzae_2. 0), *Onthophagus taurus* (GCA_000648695.2 Otau_2.0), *Dendroctonus ponderosae* (GCA_000355655.1), and *Tribolium castaneum* (GCA_000002335.3 Tcas5.2).

### 2.5. Biological Sample Collection and Preparation

The experiments were carried out on biological samples (*L. decemlineata* adults, larvae, and eggs) collected from private potato fields free from treatment by biological insecticides (Novosibirsk region, Russian Federation; 53°44′3.534″N, 77°39′0.0576″E). Due to these potato fields not being located in protected areas, there was no need for special permission to collect beetles. The landowners did not prevent access to the fields. Endangered or protected species were not used in this work. The insects were kept in a ventilated laboratory room for a 12:12 h dark/light period at a constant temperature of 25 °C. The insects were fed with fresh *Solanum tuberosum* foliage. Fourth-instar larvae (2–4 h postmolt in IV instar) were used for cuticle and hemolymph sample collection. The cuticle was cleared of the fat body in a cold phosphate buffer and frozen in liquid nitrogen, and each sample contained the cuticles of six insects. Hemolymph samples were collected as follows. A puncture was made with a sterile needle, and 25 µl of hemolymph was taken. The samples were cooled on ice and frozen in liquid nitrogen, with each being taken from a single insect [27]. For a more detailed description of biological sample collection and preparation, one can refer to the following papers [28,29].

Colorado potato beetle egg samples were prepared as follows. Ten eggs from three clutches were pulled in a single 1.5 ml Eppendorf tube (one sample contained thirty eggs). Then, 0.5 ml of 6% hydrogen peroxide was added to the sample, the sample was stirred for 5 minutes at 500 rpm on a TS-100C (BioSan) shaker, and the free liquid was removed. Then, 0.5 ml of autoclaved water was added to the sample, the tube was inverted several times, and the remaining liquid was removed with a sterile filter paper. The procedure was then repeated with 2.5% sodium hypochlorite. All manipulations were performed at room temperature with room temperature reagents, with the shaker was used without heating. The prepared samples were frozen in liquid nitrogen. This method was adapted from Rotskaya et al. [27].

### 2.6. Confirmation of the Presence of Bracovirus Genetic Fragments in the Genetic Material of L. decemlineata

For the two fragments classified as bracovirus fragments, the oligonucleotide primers were designed using Primer3 software [30]. The sequences of the fragments and primers are presented in the supplementary materials in Supplementary Table S2.

DNA was extracted from 20 mg of sterile eggs, cuticle, hemolymph, and whole insect (imago) of the Colorado potato beetle using the PureLink genomic DNA mini kit (Thermo Fisher Scientific, Waltham, MA, USA). The amplification reaction was performed in a GeneAmp PCR System 9700 thermal cycler (Thermo Fisher Scientific, USA) in a volume of 50 µL. The reaction mixture contained Taq-DNA polymerase buffer (Thermo Fisher Scientific, USA), 1.5 mM MgCl2, 0.2 mM dTTP, 0.2 mM dGTP, 0.2 mM dATP, 0.2 mM dCTP, 10 pmol of each oligonucleotide primer, 1.25 Taq-DNA polymerase activity units (Thermo Fisher Scientific, USA), and 2–10 ng matrix DNA. The amplification was performed for 30 cycles using a stepwise program (94°, 45 s; 55°, 45 s; and 72°, 2 min). The PCR products were purified using a QIAquick PCR Purification Kit (250) (QIAGEN, Germany). The amplification products were separated in a 1.2% agarose gel. The fragments obtained were confirmed using Sanger sequencing. The reaction was performed in a GeneAmp PCR System 9700 thermal cycler (Thermo Fisher Scientific, USA) in a volume of 5 µL. The reaction mixture contained prepurified DNA matrix, 2 μL of BigDye-v.3.1 sequencing solution (Thermo Fisher Scientific, USA), and a 4 pM oligonucleotide primer for 30 cycles using a stepwise program (94°, 20 s; 50°, 20 s; and 60°, 4 min).

## 3. Results

### 3.1. Analysis of the k-Mer Spectra

**Figure 2:**
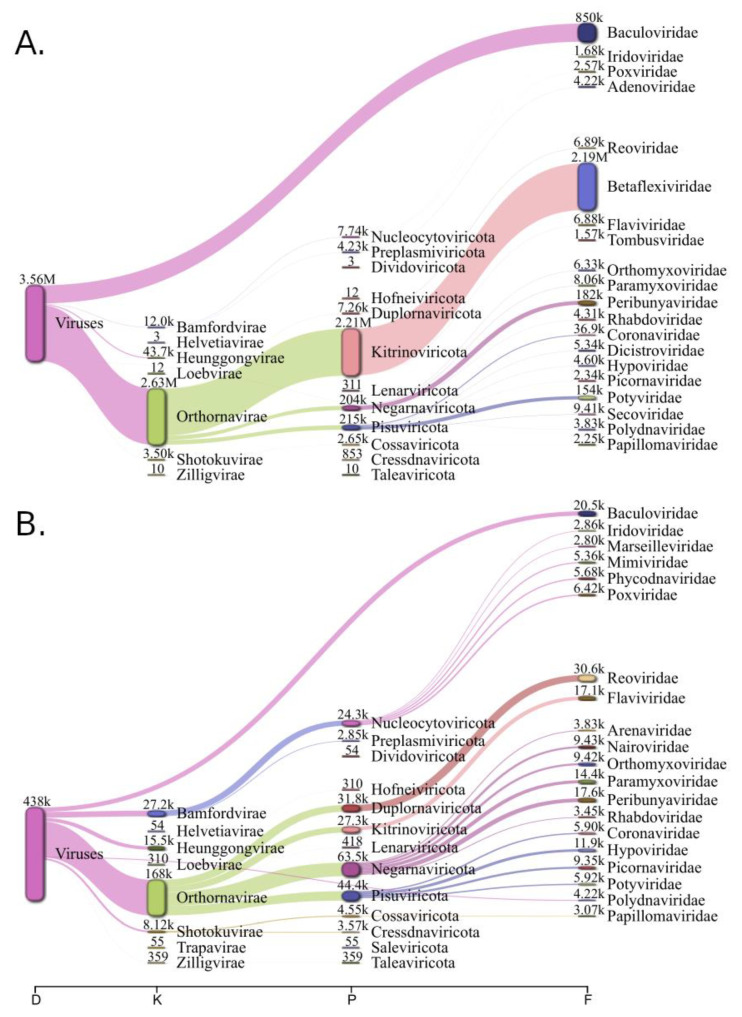
Results of k-mer spectra analysis. The classification of reads attributed as viral was performed down to the family level. The aggregated transcriptomic data analysis is shown on the top chart (**A**) and the genomic data analysis on the bottom (**B**). The numbers of reads are shown matched at each level.

The genomic and transcriptomic data of 297 *L. decemlineata* samples were analyzed in this study. The quality and contamination control of the reads for each sample resulted in no more than 5% of the reads being removed. Up to 10% of the reads were not aligned with the *L. decemlineata* genome. Among the reads not aligned with the *L. decemlineata* genome, 1.36% of the reads from the genomic samples (8,461,508 of 620,747,359) and 3.04% of the reads from the transcriptomic samples (5,905,253 of 193,959,828) were analyzed using Kraken2 and our custom BigViralDB database. The results of the analysis down to the family level are visualized in Figure 2. In the transcriptomic samples, the viral reads were classified as mainly belonging to the viral families of *Betaflexviridae, Baculoviridae, Peribunyaviridae, Potyviridae, Orthomyxoviridae, Paramyxoviridae, Dicistroviridae, Polydnaviridae*, and others. In contrast to the transcriptomic data, the genomic data lacked the reads attributed to viral families of *Betaflexviridae, Potyviridae*, and *Dicistroviridae*. The sequences attributed as retroviral or bacteriophage-related are not represented in the plots.

### 3.2. Contig Analysis

A total of 31,069,568 contigs were collected from all samples as a result of the assembly of reads unaligned to the *L. decemlineata* genome. Contig filtering by length and coverage resulted in the selection of 122,506 contigs (with an average length of 1861 bp and maximum contig length of 618,970 bp), which were further classified using BLAST tools. The final table (Supplementary Table S3) with all the results of contigs annotated with BLASTx contains 5,175,489 lines (the e-value < 10^−3^ was chosen as the filtering criterion; matches with bacteriophage proteins were excluded from the analysis). This table provides information on 13,314 unique contigs, with the superkingdom Eukaryota accounting for 13,113 unique contigs, the superkingdom Bacteria accounting for 6220, Archaea accounting for 269, and viruses accounting for 3137 contigs. A total of 719 contigs annotated with BLASTn as viral were then annotated with BLASTx. Among the contigs that could not be annotated with BLASTn, 3395 contigs were assembled from Colorado potato beetle transcriptomic reads and 9203 from genomic reads, with these then being annotated with BLASTx. 

The following tables were created based on the results of BLASTx annotation:

- Among the viral protein BLASTx hits for which the virus family was known, the best match was selected for each contig based on the e-value (minimum) and the bitscore (maximum)—2954 rows (Supplementary Table S4);
- Among the viral protein BLASTx hits for which the virus family was unknown, the best match was selected for each contig based on the e-value (minimum) and the bitscore (maximum)—1662 rows (Supplementary Table S5);
- Among the non-viral protein BLASTx hits, the best match was selected for each contig based on the e-value (minimum) and the bitscore (maximum)—13,252 rows (Supplementary Table S6).

**Figure 3:**
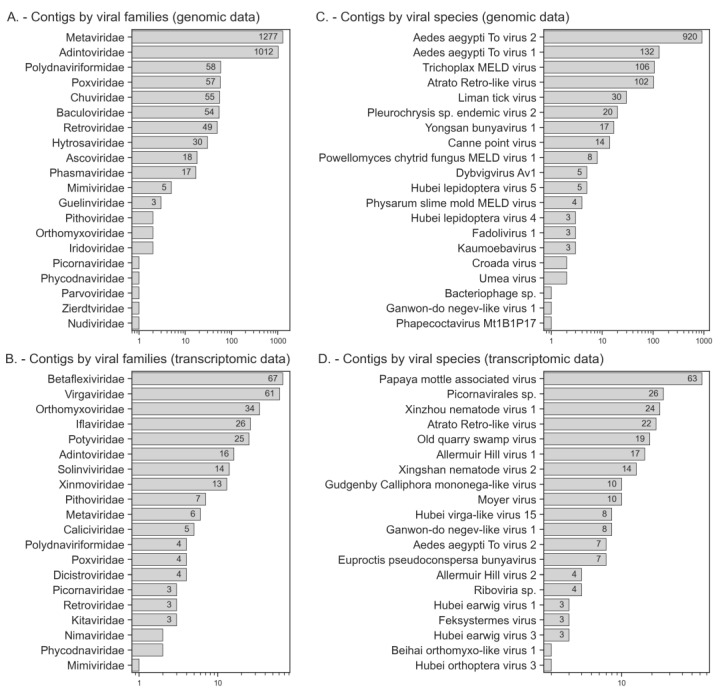
The number of virus-positive contigs by virus family in genomic (**A**) and transcriptomic (**B**) samples. The distribution of virus-positive contigs by virus species (unknown family) in the genomic (**C**) and transcriptomic (**D**) samples.

Supplementary Table S7 presents the results of the virus-positive contig annotation after filtering, including 1652 contigs assigned to different virus species with an unspecified virus family and 2954 contigs with an established virus family (with the information on contigs the protein products of which have homology to bacteriophage proteins that were excluded). The table also provides the identifiers of genomic and transcriptome data projects, the information on protein products, the distribution of contig and alignment lengths, identity scores, and e-value values. Table 1 presents the most interesting results, specifically virus-positive contigs belong to six virus types: Artverviricota, Kitrinoviricota, Negarnaviricota, Nucleocytoviricota, Pisuviricota, and Preplasmiviricota, distributed in 17 orders, 32 viral families, and 97 species. In addition, at least 48 virus species with unknown viral families were also matched. Figure 3 shows the distributions of virus-positive contig numbers derived from genomic and transcriptomic samples by virus family or species (if family is unknown). If homology with viral sequences was not taken into account, then most of these contigs were attributed to insects (Coleoptera), as is shown in Supplementary Figures S1 and S2.

The highest number of annotated virus-positive contigs belonged to the endogenous viral elements of retroviral origin: species of *Aedes aegypti To virus 2* (*AAToV2*), *Aedes aegypti To virus 1* (*AAToV1*), and *Atrato Retro-like virus* with unspecified taxonomic affiliations and a number of virus representatives of the families *Metaviridae* and *Retroviridae.* The identity percentage ranges from 31.5% to 85.7% and is higher for the members of the *Metaviridae* family, *AAToV1* and *AAToV2*. For *Trichoplusia ni TED virus (Metaviridae)*, the maximum alignment length reaches 1157 aa. More than 1000 contigs obtained mainly from genomic data were annotated as belonging to different members of the family *Adintoviridae*. The alignments indicate homology not only in terms of retrovirus-like integrase and polymerase B but also in terms of structural proteins of the family *Adintoviridae*.

Many virus-positive contigs were found to be associated with viruses infecting insects. Twenty-six contigs with a similarity to *Lampyris noctiluca iflavirus 1* (family *Iflaviridae*) were identified. The maximum length of alignments between the proteins encoded in these contigs and the polyprotein of this virus was 1642 aa (median 1638 aa), with a median identity of 33.8% (maximum identity percentage 39.7%). The genome size of the *Iflaviridae* family members varies from 9 to 11 kb, with a median length among the corresponding virus-positive contigs of 9870 nucleotides (maximum 9934). Two of the four contigs assigned to the family *Dicistroviridae* were attributed to the species *Aphis gossypii virus* and *Aphid lethal paralysis virus*, with the contig lengths being 6635 and 3468 nucleotides, respectively, and the genome sizes of representatives of the family *Dicistroviridae* ranged from 8 to 10 kb. The percentage of alignment identity predicted for these contigs is 93.9% for the ORF1 protein (alignment length 1039 aa) and 92.8% for the capsid protein (alignment length 808 aa). Along with the families *Iflaviridae* and *Dicistroviridae*, other virus-positive contigs homologous to the genetic sequences of the order Picornavirales were assigned to insect-infecting members of the *Picornaviridae, Solinviviridae*, and *Caliciviridae* families. The identity percentage of the proteins encoded by these contigs versus the polyproteins of these virus families does not exceed 30%. Many of these contigs are extended and range from 3350 to 11648 nucleotides in length. The protein products of 36 contigs annotated as belonging to insect-infecting members of the family *Orthomyxoviridae* (*Photinus pyralis orthomyxo-like virus 1, Quaranjavirus araguariense, Coleopteran orthomyxo-related viruses*, etc.) showed homology with various structural and nonstructural proteins, with identities of 25 to 55% and alignment lengths reaching 768 aa. 

A total of 55 contigs derived from genomic samples were annotated as belonging to the family *Baculoviridae*, with most contigs encoding polypeptides homologous to F-protein (with a maximal alignment length of 474 aa and maximal identity of 30.9%). In addition, translation frames were identified, with their products homologous to vp80 (156 aa alignment length, 35.9% identity), ORF27 (220 aa alignment length, 26.8% identity), and PxORF26 (271 aa alignment length, 21% identity). Additionally, more than 70 contigs containing translation frames homologous to glycoproteins from various members of the families *Chuviridae* and *Phasmaviridae* were also detected. The identity of the encoded amino acid sequences to the Coleopteran chu-related virus glycoprotein OKIAV127 (family *Chuviridae*) was as high as 72.8%. A total of 62 contigs up to 6 kb in length were identified, with their predicted proteins being homologous with various amino acid sequences of representatives of the genus *Bracoviriform* (family *Polydnaviriformidae*). Additionally, a contig with a length of 17638 nucleotides encoding a protein 28.6% identical to the protein P74 of *Penaeus monodon nudivirus* (family *Nudiviridae*) was found, with an alignment length of 655 aa.

Additionally, we also identified contigs attributed to plant viruses. Within 66 extended contigs exceeding 8 kb in length, translation frames encoding proteins with 100% identity to *Potato virus S* (family *Betaflexiviridae*) were found, with an alignment length of 1975 aa. A contig was found with a protein product 99.6% homologous to the *Potato virus H* (family *Betaflexiviridae*) shell protein, with an alignment length of 292 aa. Twenty-five contigs longer than 9 kb were found to be assigned to *Potato virus Y* (family *Potyviridae*), with an alignment length of 3061 aa (polyprotein). Given that the genomes of these potato viruses range from 5.4 kb to 12 kb in length, we obtained almost complete genome assemblies. In addition, we identified 63 extended (up to 8.5 kb) contigs belonging to the *Papaya mottle associated virus*, with their protein products being homologous to RdRp of the virus specified (maximal identity was 64.4%, maximal alignment length was 1017 aa). Additionally, we identified single contigs of plant viruses with protein products homologous to those of *Garlic common virus*, *Bromoviridae*, and *Kitaviridae* (with identity less than 50%, and the alignment length less than 200 aa). 

In addition, contigs were found encoding protein products homologous to viral proteins of the replication system, such as RdRp, and to uncharacterized viral proteins, with identities of 30–40% and alignment lengths not exceeding 300 aa. In particular, 19 contigs identified in the study were attributed to *Trichoplusia ni ascovirus 2c* (family *Ascoviridae*), with the alignment of the identity of the encoded protein products to various uncharacterized viral proteins being 40.6% and the maximum alignment length being 137 aa. In the genomic samples, we also identified two contigs whose protein products were 31% identical to the short amino acid sequences, 168 aa in length, of uncharacterized *Iridovirus LCIVAC01* proteins (family *Iridoviridae*). Extended contigs of more than 10 kb in length were also detected, with alignment lengths of 1930 aa and with them being 30% identical to the RdRp of *Xinmoviridae* family representatives. Extended contigs encoding proteins homologous to RdRp, ORF3, and hypothetical proteins of the *Virgaviridae* family were also identified. Moreover, several contigs exhibited homology in terms of their protein products to uncharacterized proteins of various representatives of the *Poxviridae, Hytrosaviridae, Partitiviradae, Bornaviridae, Rhabdoviridae, Parvoviridae* families and several viruses of unknown viral families. 

**Figure 4:**
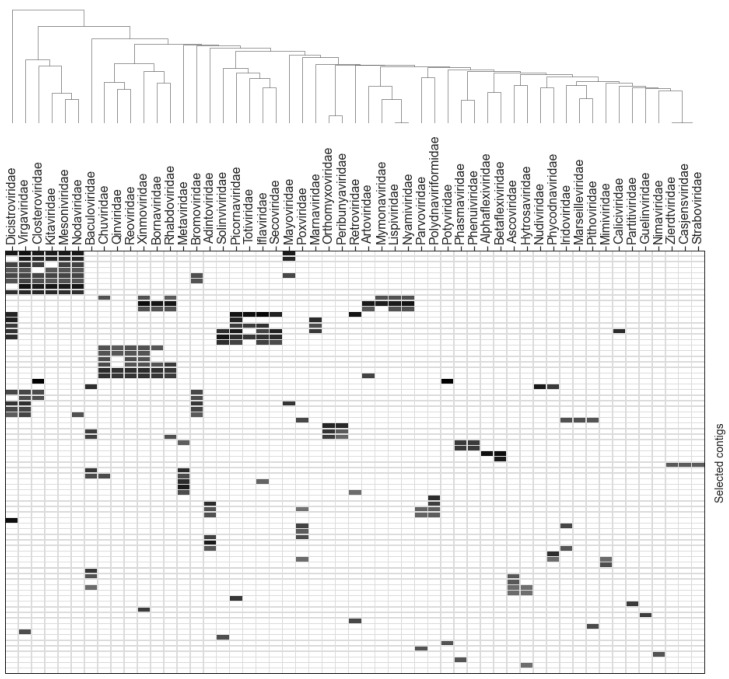
Clustering of selected contigs and viral families based on the homology of contig-encoded proteins with proteins from different virus families. The color density is proportional to the decimal logarithm of overall alignment length with corresponding viral family according to BLASTx results. The white color corresponds to the lack of significant alignments.

Protein products encoded by the same contig tend to exhibit significant homology with related viruses. To understand how viral annotations are related to each other, we additionally clustered contigs based on the homology of the encoded proteins with proteins from different viral families (Figure 4). The maximum alignment length of the proteins encoded by each contig was calculated for each contig relative to the proteins of each of the presented viral families. The alignment lengths of the proteins encoded by each of the contigs with the amino acid sequences of each of the viral families are presented as values in the heatmap cells. Figure 4 shows the contigs with only a unique homology pattern. 

It can be seen that contigs are clustered largely based on the phylogenetic kinship of viral groups. In particular, contigs with proteins homologous to those of viruses of the *Dicistroviridae* family are clustered with RNA-containing viruses, including other families of the Picornavirales order (*Picornaviridae, Iflavirudae, Secoviridae, Caliciviridae,* and *Marnaviridae*) as well as with virus families of the Martellivirales order (*Virgaviridae, Kitaviridae,* and *Closteroviridae*). Contigs encoding proteins that are homologous to proteins of phylogenetically close orders, such as Mononegavirales (*Xinmoviridae, Bornaviridae,* and *Rhabdoviridae*) and Jingchuvirales *(Qinviridae* and *Chuviridae*), are also clustered. A separate cluster is formed by contigs encoding proteins homologous to those of viruses of the families *Xinmoviridae, Bornaviridae, Rhabdoviridae, Artoviridae, Mymonaviridae, Lispiviridae,* and *Nyamiviridae* of the order Mononegavirales. A number of contig clusters encode proteins that are homologous to proteins of Nucleocytoviricota representatives (families *Iridoviridae, Ascoviridae, Phycodnaviridae, Pithoviridae, Mimiviridae,* and *Marseilleviridae*). A full version of the table containing the data used in Figure 4 is presented in Supplementary Table S8. An interactive visualization of all the contigs analyzed can be found at the project repository at https://github.com/starchevskayamaria17/uncoVir, accessed on December 2022. Most contigs whose protein products were not classified as viral proteins were found to have homology with the amino acid sequences of arthropods and mainly with insect proteins.

### 3.3. Non-Viral Protein Hits Are Significantly Enriched with Unknown and Uncharacterized Proteins as Compared to Viral Protein Hits

**Figure 5:**
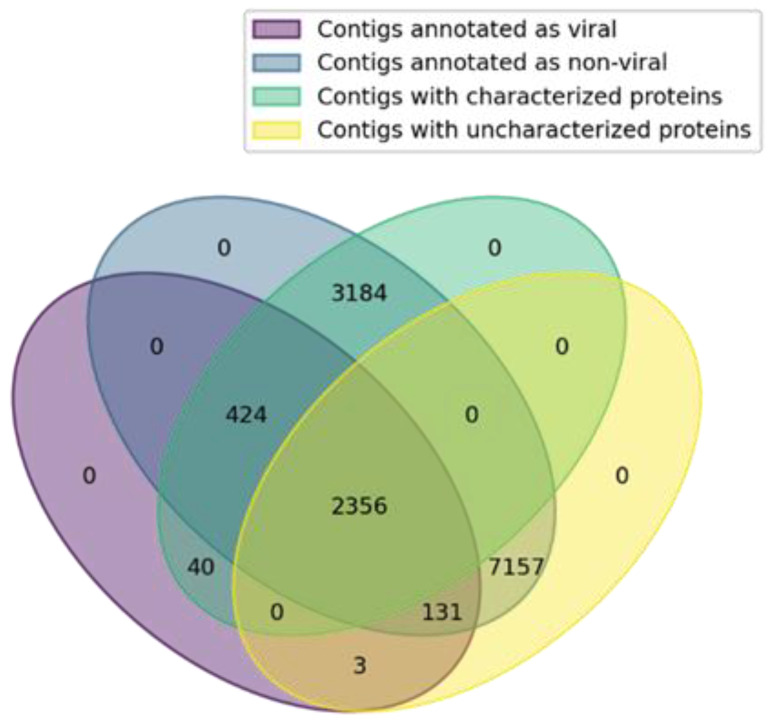
Venn diagram illustrating the distribution of contigs into groups: viral/non-viral and with known/unknown proteins. The number of contigs with “unknown” proteins among non-viral contigs exceeds the number among viral contigs.

The next step was to analyze the enrichment of the contigs under study with unknown and uncharacterized proteins. The statistical analysis was performed using Fisher’s exact test for unpaired sets (nonoverlapping sets of contigs) and the McNemar test for paired sets (contigs whose proteins have homologs among both viral and non-viral proteins). The entire set of identified homologous proteins was divided into the following groups: “viral” and “non-viral” and “known” and “unknown.” Proteins with the following keywords in their name were considered unknown: unnamed, uncharacterized, unknown, or hypothetical. The set of contigs were also categorized into groups: “viral” and “non-viral” contigs whose proteins have and lack homology with viral proteins, respectively (Figure 5). 

The analysis was performed for all “viral” contigs, which were found to be homologous with any of the viral families, and “non-viral” contigs, whose proteins were found to be homologous only with non-viral proteins. A total of 2817 “known” and 137 “unknown” proteins were found for “viral” contigs, and 3144 and 7033 were found for “non-viral” contigs, respectively. Thus, the enrichment with “unknown” proteins among the non-viral proteins was shown to be significant (*p* < 10^−6^, one-way Fisher’s exact test).

We also performed unknown protein enrichment analysis for contigs with proteins shown to be homologous with viral and non-viral proteins. In this case, 2817 “known” and 137 “unknown” proteins were found among “viral” proteins, and 427 and 2484 were found among “non-viral” proteins, respectively. Thus, the “unknown” protein enrichment among non-viral proteins was shown to be significant (*p* < 10^−6^, one-way McNemar test). This finding can be explained by the fact that many eukaryotic genes encoding uncharacterized and hypothetical proteins may be of viral origin. 

### 3.4. Alignment Results

The mapping of reads to viral genomes and the extraction of consensus sequences yielded the near full-length or extended genomic sequences for a number of viruses. Table 2 shows the sizes of the reference genomes and the most extended consensus sequences (in nucleotides and % relative to the reference size). Extended information about the alignments is provided in Supplementary Table S9. 

The sequences representing 99.63% and 99.95% of the corresponding reference genomes were obtained for *Potato virus Y* (family *Potyviridae*) and *Potato virus S* (family *Betaflexiviridae*), respectively. The size of the consensus sequences obtained as a result of the alignment for five members of the family *Dicistroviridae* ranged from 10.39% to 70.84% of their genome size. Additionally, the alignment allowed us to obtain the consensus sequences of genome fragments of other viruses known to affect insects, such as the *Wuhan insect virus 33*, and the representatives of the families *Virgaviridae, Baculoviridae*, and *Polydnaviriformidae*.

### 3.5. Analysis of Selected Coleopteran Species Reference Genomes to Detect Bracoviral Genetic Fragments

**Figure 6:**
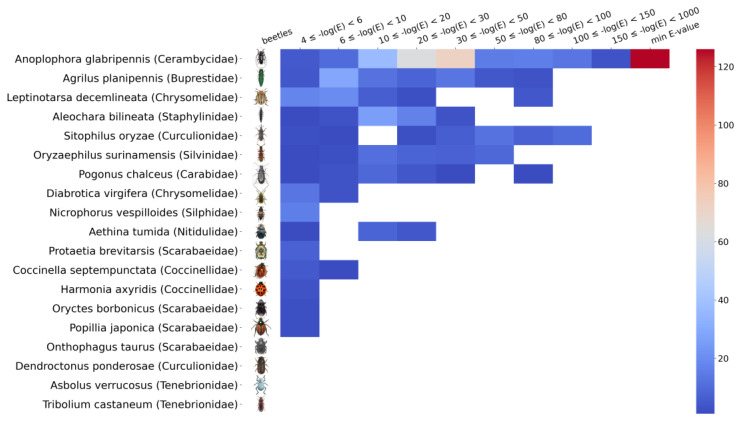
Distribution of bracovirus k-mers depending on e-value in reference genomes of several members of Coleoptera, including *Leptinotarsa decemlineata*. The color indicates the number of bracovirus k-mers identified within the specified e-value interval (BLASTn short). The absence of the color designates the absence of a match.

The k-mer analysis and the contigs annotation revealed numerous sequences belonging to members of the genus *Bracoviriform* (family *Polydnaviriformidae*). The analysis of the reference genome *Leptinotarsa decemlineata* revealed numerous sequences homologous to the genus *Bracoviriform*, with a total length of approximately 25 kbp. Several other coleopteran genomes were also analyzed. Figure 6 shows a heatmap of the distribution of k-mers classified as *Bracoviriform* depending on the e-value.

Two sequences with homology to the segments of the bracovirus genomes were selected for the subsequent experimental studies. The nucleotide sequences of contigs 1513 and 2100 nucleotides long were homologous to *Cotesia sesamiae Mombasa bracovirus* (EF710639.1, the coordinates of the reference sequence alignment: 57046–58557, identity percentage 100%) and *Cotesia vestalis bracovirus* segment 35 (HQ009558.1, the coordinates of the reference sequence alignment: 1797–3455, identity percentage of 98.13%), respectively. These contigs were also found to be aligned to the *Leptinotarsa decemlineata* genome. The first bracovirus contig was aligned to NW_019290920.1, and the coordinates of the reference sequence alignment were 2663–4169; the second contig was aligned to NW_019296350.1, and the coordinates of the reference sequence alignment were 9973–11595. The alignment allows us to assume the insertion of viral DNA of *Bracoviriform* genus into the genome of *Leptinotarsa decemlineata*.

We calculated the primers so as to confirm the presence of bracovirus fragments in the genetic material of *Leptinotarsa decemlineata*, to prove the insertion into the *Leptinotarsa decemlineata* genome, and to exclude the presence of fragments containing regions flanking the insertion in the corresponding bracovirus genome. The PCR analysis of DNA obtained from different tissues of *Leptinotarsa decemlineata* (eggs, sterile eggs, cuticle, hemolymph, and whole imago insect) confirmed the presence of both bracovirus fragments and revealed the absence of fragments containing the regions flanking the insertion in the bracovirus genome. The nucleotide sequence of the fragments was confirmed by Sanger sequencing. The insertion into the *Leptinotarsa decemlineata* genome was not confirmed by this experiment, which may be due to assembly errors or high variability of corresponding regions of the *L. decemlineata* genome (Supplementary Figures S3–S6).

## 4. Discussion

Among the annotated sequences, the contigs of viral origin can be divided into several groups: those homologous to genetic sequences of insect viruses (representatives of the families *Bornaviridae, Caliciviridae, Dicistroviridae, Iflaviridae, Iridoviridae, Orthomyxoviridae, Partitiviridae, Picornaviridae, Poxviridae,* and *Virgaviridae, Xinmoviridae*), plant viruses (representatives of the families *Betaflexiviridae, Bromoviridae, Kitaviridae,* and *Potyviridae*), endogenous retroviral elements (*Retroviridae* and *Metaviridae*), endogenous viral elements of non-retroviral origin (*Adintoviridae, Ascoviridae, Chuviridae, Hytrosaviridae, Nimaviridae, Nudiviridae, Phasmaviridae, Polydnaviriformidae, Rhabdoviridae,* and *Baculoviridae*), bacteriophage sequences and protozoan viruses (not described within this study), and numerous viral sequences of unknown family primarily affecting insects. 

### 4.1. Insect Viruses

Numerous sequences of insect-infecting viruses were found in the data analyzed. Special attention should be given to the viruses of the families *Iflaviridae* and *Dicistroviridae* (the members of the order Picornavirales), whose genetic sequences were detected at different stages of the analysis. The members of the families in question are characterized by a rather small ss(+)RNA genome measuring 8–10 kb for the family *Dicistroviridae* and 9–11 kb for the family *Iflaviridae*. It is worth noting that new virus species of these families are often identified by metagenomic analysis in the NGS data of various insects, particularly mosquitoes [31]. The *Homalodisca coagulata dicistrovirus* also identified in this way is the virus of a glassy-winged sharpshooter *Homalodisca coagulata* (Cicadellidae), which feeds on hundreds of plant species and carries plant pathogens, causing enormous damage to agriculture [32]. Here, we obtained numerous sequences attributed to these two families of viruses, with contig lengths commensurate with their complete genome lengths, some of which are likely the genetic sequences of new viruses of these families. The contigs annotated as homologous to *Lampyris noctiluca iflavirus 1* reach a length of 10 kb. However, the amino acid sequence of the polyprotein encoded by these contigs is only 30% identical to that of the iflavirus. In this study, we identified sequences homologous to *Homalodisca coagulata dicistrovirus* (comprising over 40% of the genome), *Aphid lethal paralysis* virus (up to 70% of the genome and an extended contig with a length of 3468 nucleotides, encoding a polypeptide that is 92% identical to the viral protein), *Aphid glycines virus 3* (over 70% of the genome), *Aphis gossypii* virus (a 6635-nucleotide-long extended contig encoding a protein product that is 93% identical to the viral polyprotein), and others. The majority of known members of the family *Dicistroviridae* have been shown to infect only a limited number of related insect species (belonging to the same order). However, there are viruses in this family that infect a vast range of hosts. For example, *Cricket paralysis virus* can infect more than 20 insect species from different orders [33].

Earlier studies suggested that the Colorado potato beetle could be infected by viruses of the *Baculoviridae* and *Iridoviridae* families [34]. Various baculoviruses are known to affect a wide variety of insect taxonomic groups. Currently, the members of the family *Baculoviridae* are used as safe and effective biopesticides to protect forests, fields, and horticultural crops against lepidopteran pests [35]. Most of the contigs found in the genomic data and annotated as baculoviruses exhibit homology to the F-protein gene of various members of the genera *Alphabaculovirus* and *Betabaculovirus* and will be described in more detail below in the “Endogenous viral elements” section. Despite a number of contigs being found to be homologous with baculovirus sequences different from the F-protein gene, the low length of such fragments did not allow us to annotate them reliably. 

Among the members of the Nucleocytoviricota type, a number of viruses of the families *Iridoviridae*, *Poxviridae*, and *Ascoviridae* are known to affect insects. However, despite the presence of contigs assigned to these families, the low percentage of identity (30–40%) and alignment length (64 to 191 aa), as well as the short length of contigs (from 500 to 2044 nucleotides), did not allow conclusions to be drawn about the presence of genetic material of specific viruses of these families in the samples analyzed. These sequences may also be fragments of endogenous viral elements integrated into the *L. decemlineata* genome over the course of evolution. The presence of endogenous viral elements of iridovirus origin in insect genomes is indicated, in particular, by the fact that certain representatives of leaf-feeding beetles, such as *Gastrophysa atrocyanea*, were shown to produce a diapause-specific peptide that acts as a blocker of potential-dependent calcium channels, provides antifungal activity, and is homologous to the sequence of the peptide encoded by *Iridoviridae*. The sequences encoding these peptides have been suggested to be suitable as probes for analyzing insects and this group of viruses [36].

For several other viruses affecting insects, such as members of the families *Ortomyxoviridae, Virgaviridae, Xinmoviridae,* and *Partiviridae*, and viruses with unspecified families, such as *Wuhan insect virus 33*, *Hubei picorna-like virus 15,* and *Allermuir Hill virus 1*, extended amino acid alignments with various structural and nonstructural viral proteins were found, with the corresponding virus-positive contigs being highly extended.

### 4.2. Plant Viruses

Plant pathogenic viruses pose a serious threat to livestock as well as agricultural, horticultural, and ornamental crops [37,38]. The *Potato virus Y* detected in this study belongs to the RNA-containing virus family *Potyvirus* of the family *Potyviridae*, which represents approximately 30% of all known plant pathogenic viruses. *Potato virus Y*, usually transmitted by aphids, can significantly reduce potato yields [39]. *Potato virus S*, the full-length genome of which was also identified in this work, belongs to the RNA-containing viruses of the *Betaflexviridae* family. Despite having less pronounced disease symptoms in the affected plants, *Potato virus S* can destroy up to 20% of crops [40]. These results confirm the presence of vast amounts of potato viruses in *L. decemlineata* tissues and especially in gut and head samples. 

In addition, leaf-feeding beetles transmit a variety of plant viruses [41,42]. Thus, *Leptinotarsa decemlineata* might also be involved in the mechanistic transmission of potato viruses. It is interesting to note that the *Potato virus Y* reduces the production of potato defense factors, such as sesquiterpenes, namely beta-barbatene, thus increasing the growth of Colorado potato beetle larvae feeding on infected plants [39], and, as a result, the plants are exposed to a combination of severe biotic stressors, increasing crop losses [39].

In addition, we identified 63 extended (up to 8.5 kb) contigs belonging to the *Papaya mottle associated virus*, with their protein products being homologous to RdRp. Additionally, we identified several plant virus-positive contigs with protein products that are homologous to those of *Garlic common virus*, *Bromoviridae*, and *Kitaviridae* with alignment lengths of less than 200 aa and identities of less than 50%. Thus, it was not possible to make any strong conclusions on these contigs.

### 4.3. Endogenous Virus Elements

The ability of many viruses to integrate their genetic material into the host genome is widely known. It was reported that 0.59% of the genome of *L. decemlineata* contains LTR regions, including Copia, Gypsy, Gypsy-Cigr, and Pao [8]. This study identified numerous retroviral sequences homologous to the members of the families *Retroviridae* and, to a greater extent, *Metaviridae*, which are characteristic to insects (LTR retrotransposon, Ty3/gypsy family), as well as sequences homologous to the unclassified viruses *AAToV1* and *AAToV2*. Along with integrated retroviral sequences, we discovered viral sequences of non-retroviral origin, called endogenous viral elements (EVEs). The currently known insect EVEs belong to at least 28 viral families [43]. The endogenization of viral sequences into the host genome and EVE formation can occur via nonhomologous recombination or via the participation of the reverse transcriptase and integrase of endogenous retrotransposons. EVEs can also contribute to the emergence of new functional host genes and even play an antiviral role by generating small RNA, thereby preventing the maturation of viral particles at various stages of the viral life cycle [44,45,46].

Recent studies of insect genomes have uncovered numerous genetic sequences of negative sense RNA-containing viruses [43,47]. Such studies confirm the fact that arthropods are one of the main reservoirs of viral genetic diversity and undoubtedly play an essential role in virus evolution. Genetic sequences of the members of the family *Chuviridae*, characterized by an unusual ordering of genes, have been found in many genomic sequences of various invertebrates [48]. In particular, integrated genes of *Chuviridae* glycoproteins were found in the genomes of *Dendroctonus ponderosae* and *Tribolium castaneum* (Coleoptera) [47]. For the mosquitoes *Aedes aegypti* and *Aedes albopictus*, the presence of endogenous Chuviridae-like genes in Bel/Pao elements (family *Belpaoviridae*) was described. In our study of genomic data samples, we have found contigs encoding proteins similar to the glycoproteins of viruses of the *Chuviridae* family, including *the Coleopteran chu-related virus*. In addition, in the genomic data of *L. decemlineata,* we also found sequences homologous to the genes of viruses of the families *Phasmaviridae, Rhabdoviridae, Xinmoviridae, Bornaviridae,* and *Orthomyxoviridae*. These viruses have previously been shown to be present as EVEs within the genomes of various insect families [43].

Of interest is the fact that sequences homologous to the baculovirus F-protein gene were found in the genomic data of *L. decemlineata*. It is believed that the ancestral baculoviruses could replicate only in the midgut epithelial cells of insects, and the F-protein gene acquired during the evolution allowed the virus to penetrate the hemocoel and cause generalized infection. Previously, the orthologs of the baculovirus F-protein gene were found in the endogenous retroviral sequences of lepidopterans, dipterans, and other insect species, indicating that the regions encoding F-protein homologs identified in our study can also be incorporated into the genome of *L. decemlineata* [49,50], with this possibly indicating that the Colorado potato beetle can also be affected by baculoviruses.

We also identified numerous extended fragments homologous to the genetic sequences of viruses of the genus *Bracoviriform*. The members of the genus *Bracoviriform* belong to the family *Polydnaviriformidae* and are associated with parasitoid wasps, including approximately 18,000 species of five subfamilies of the family Braconidae (Microgastrinae, Cardiochilinae, Miracinae, Khoikholinae, and Cheloninae). The genomes of *Polydnaviriformidae* family viruses are segmented; their segments have multiple copies and present in virions non-equimolarly in the form of circular supercoiled double-stranded DNA measuring from 2 to 31 kbp. The cumulative non-redundant size of the bracovirus genome varies from 190 to 500 kbp [51]. The viral genome is transmitted as proviral DNA in parasitoid wasp cells and circular episomal DNA within virions. The replication of the virus genome and virion assembly occurs in ovarian cells, with viral particles being released and accumulated in the lumen of the oviduct and entering the host larvae or eggs during wasps laying their own eggs. In other wasp cells, the virus does not replicate and exists predominantly in a proviral form. Interestingly, the expression of viral genes causes changes in the physiology of the infected host and suppresses the encapsulation of wasp eggs, promoting the development and emergence of wasps [52]. Wasps parasitize on the larvae and eggs of lepidopterans, coleopterans, and hymenopterans. Despite the absence of replication in the cells of lepidopterans, the integration of bracovirus genome segments has been demonstrated both in vitro, in cell cultures, and in vivo [51]. Certain bracovirus genes were shown to contribute to protecting insects against baculoviruses—lethal pathogens, which explains why these genes have been conserved in the genomes of many lepidopterans [53].

We found extended bracovirus sequences in the reference genome of *L. decemlineata* and in the genomes of several other representatives of Coleoptera. We also demonstrated the presence of bracovirus sequences in the genetic material isolated not only from imago and larva insect tissues but also from sterile eggs of *L. decemlineata*, suggesting the integration of bracovirus genomic sequences within the Colorado potato beetle genome. To our knowledge, we are the first to report on the detection of bracovirus sequences in the genomes of the Colorado potato beetle and leaf-feeding beetles. The presence of these fragments in the Colorado potato beetle genome also appears to be associated with parasitoid wasps, such as the wasp *Edovium puttleri,* which parasitizes the eggs of *Leptinotarsa decemlineata* [54].

Additionally, the other sequences identified in our study, which are homologous to the genetic sequences of *Nudiviridae* family viruses, are also likely associated with bracovirus genetic fragments, given that the genus *Bracoviriform* originated from the *Nudiviridae* family [55].

Thus, the metagenomic studies of accumulated public high-throughput sequencing data serve as an important source of information for the search for new viruses. This work highlights the significance of metagenomic studies of genomic and transcriptomic data obtained from insect pests for a better understanding of their ecology and evolution. The discovery of genetic sequences potentially belonging to insect-infecting viruses points to the possibility of expanding the arsenal of biological methods to control the Colorado potato beetle population based on entomopathogenic viruses.

## Figures and Tables

**Table 1:** The number of contigs by viral species, family, and order, with maximum alignment length, alignment identity percentage, and maximum contig length. The complete table is given in Supplementary Table S7 with project identifiers, protein products, extended taxonomy, and alignment e-values, etc.

Order	Family	Species	Max Alignment Length, aa	Max Identity, %	Max Contig Length, bp	Number of Contigs	Protein Product
Martellivirales	*Virgaviridae*	*Nephila clavipes virus 4*	1047	40.51	10,559	51	RdRp
		*Broome virga-like virus 1*	177	31.64	2499	5	ORF3
		*Insect virga-like virus 1*	165	41.67	540	2	Hypothetical protein
Tymovirales	*Betaflexiviridae*	*Potato virus S*	1975	100	8539	66	Structural and nonstructural polyprotein
Bunyavirales	*Phasmaviridae*	*Wuhan mosquito orthophasmavirus 1*	388	27.06	1667	17	Glycoprotein
Articulavirales	*Orthomyxoviridae*	*Quaranjavirus araguariense*	437	27.82	1718	9	Hemagglutinin
		*Photinus pyralis orthomyxo-like virus 1*	768	43.94	2529	15	Nucleocapsid protein, polymerase PB2
		*Coleopteran orthomyxo-related virus OKIAV184*	501	31.34	1715	2	Hemagglutinin
		*Coleopteran orthomyxo-related virus OKIAV186*	761	46.78	2607	2	Polymerase PB2
Jingchuvirales	*Chuviridae*	*Nigecruvirus ixodes*	631	36.42	2176	14	Glycoprotein
		*Coleopteran chu-related virus OKIAV127*	167	72.79	1926	22	
		*Orthopteran chu-related virus OKIAV152*	138	38.41	2075	1	
Mononegavirales	*Xinmoviridae*	*Orthopteran anphevirus*	383	25.10	1645	3	Hypothetical protein 1
		*Ulegvirus freckenfeldense*	1930	30.95	14,588	9	RdRp
Pimascovirales	*Ascoviridae*	*Trichoplusia ni ascovirus 2c*	137	40.63	1321	19	Hypothetical protein
Pimascovirales	*Iridoviridae*	*Iridovirus LCIVAC01*	168	30.95	796	2	Uncharacterized protein
Picornavirales	*Caliciviridae*	*Soybean thrips Pernambuco virus*	497	25.81	11,259	6	Polyprotein
	*Dicistroviridae*	*Aphid lethal paralysis virus*	808	92.82	3468	1	Capsid protein
		*Aphis gossypii virus*	1039	93.94	6635	1	ORF1
		*Bivalve RNA virus G5*	369	24.93	6436	1	Replicative protein
	*Iflaviridae*	*Lampyris noctiluca iflavirus 1*	1642	39.73	9934	26	Putative polyprotein
	*Solinviviridae*	*Solenopsis invicta virus 3*	966	29.12	11,648	12	Structural and nonstructural polyprotein
Patatavirales	*Potyviridae*	*Potato virus Y*	3061	100	9728	25	Coat protein, polyprotein
Lefavirales	*Baculoviridae*	*Mythimna unipuncta nucleopolyhedrovirus*	309	25.21	1929	14	F-protein
		*Peridroma alphabaculovirus*	474	30.95	2668	9	
		*Mythimna unipuncta granulovirus B*	251	25.10	1628	14	
		*Helicoverpa armigera nucleopolyhedrovirus*	276	22.60	1550	4	Envelope protein, hypothetical protein
		*Lambdina fiscellaria nucleopolyhedrovirus*	156	35.90	962	1	Vp80
		*Plutella xylostella granulovirus*	271	21.03	1275	1	PxORF26 peptide
		*Xestia c-nigrum granulovirus*	220	26.82	1394	1	ORF27
	*Nudiviridae*	*Penaeus monodon nudivirus*	655	28.55	17,638	1	P74
	*Polydnaviriformidae*	*Bracoviriform congregatae*	242	71.43	1669	14	Structural and nonstructural protein
		*Bracoviriform inaniti*	419	43.46	2975	28	
		*Cotesia sesamiae bracovirus*	614	54.76	6071	19	
		*Bracoviriform facetosae*	158	25.95	716	1	
		*Wuhan insect virus 33*	1039	99.32	6635	1	Hypothetical protein
		*Papaya mottle associated virus*	1017	64.40	8539	63	RdRp
		*Old quarry swamp virus*	794	46.36	2607	20	Putative nucleocapsid, polymerase
		*Allermuir Hill virus 1*	1016	46.63	9840	17	Putative nonstructural polyprotein

**Table 2:** Characteristics of reads’ alignments to viral genomes. This table specifies the family, genus, and species of the virus, genome coverage information (maximum, average, and median, in nucleotides and percentages), the minimum and maximum genome lengths stored in the BigViralDB database for the specified viral family, and the number of *L. decemlineata* genomic and transcriptomic projects where these viral sequences were found.

Family	Species	Coverage, Bases			Coverage, %			Genome Length		Projects
		Min	Max	Median	Min	Max	Median	Min	Max	
*Potyviridae*	*Potato virus Y*	55	9561	100.50	0.57	98.63	1.03	9595	9800	8
*Betaflexiviridae*	*Potato virus S*	55	8481	6336.00	0.65	99.95	74.56	8453	8515	10
*Dicistroviridae*	*Aphis glycines virus 3*	133	6691	3412.00	1.31	70.84	36.08	9445	10,131	2
	*Aphid lethal paralysis virus*	6635	6635	6635.00	67.57	67.57	67.57	9819	9819	1
*Betaflexiviridae*	*Potato virus H*	6520	6520	6520.00	77.53	77.53	77.53	8410	8410	1
*Unclassified*	*Wuhan insect virus 33*	5877	5877	5877.00	57.44	57.44	57.44	10,232	10,232	1
*Dicistroviridae*	*Homalodisca coagulata virus 1*	3998	3998	3998.00	42.78	42.78	42.78	9345	9345	1
*Retroviridae*	*Human endogenous retrovirus K*	90	2908	277.00	0.95	30.70	2.92	9472	9472	6
*Virgaviridae*	*Turnip vein-clearing virus*	2795	2795	2795.00	44.29	44.29	44,29	6311	6311	1
*Herpesviridae*	*Human betaherpesvirus 5*	50	2647	191.00	0.02	1.15	0.08	229,209	235,732	14
*Togaviridae*	*Semliki Forest virus*	100	1838	622.00	0.69	12.65	4.28	14,529	14,529	12
*Unclassified*	*Aphis glycines virus 2*	1567	1567	1567.00	32.31	32.31	32.31	4850	4850	1
	*Hubei picorna-like virus 15*	1395	1395	1395.00	14.08	14.08	14.08	9910	9910	1
*Dicistroviridae*	*Rhopalosiphum padi virus*	1235	1235	1235.00	12.29	12.29	12.29	10,045	10,045	1
*Unclassified*	*Wuhan aphid virus 1*	1185	1185	1185.00	41.89	41.89	41.89	2829	2829	1
*Baculoviridae*	*Autographa californica multiple nucleopolyhedrovirus*	50	1086	294.00	0.04	0.92	0.23	118,582	138,991	8
*Dicistroviridae*	*Big Sioux River virus*	1064	1064	1064.00	10.39	10.39	10.39	10,237	10,237	1
*Paramyxoviridae*	*Canine morbillivirus*	105	1017	453.00	0.56	5.40	2.41	18,826	18,826	12
*Adenoviridae*	*Human mastadenovirus C*	55	775	182.00	0.15	2.16	0.51	35,926	35,958	5
*Polydnaviriformidae*	*Chelonus inanitus bracovirus*	368	722	545.00	1.72	3.38	2.55	21,376	21,376	2
*Flaviviridae*	*Pestivirus A*	36	401	92.50	0.25	3.16	0.68	12,295	15,752	14
*Pospiviroidae*	*Citrus exocortis viroid*	121	376	214.00	30.56	94.95	54.04	396	396	13
*Polydnaviriformidae*	*Diolcogaster facetosa bracovirus*	73	345	101.00	0.07	0.98	0.10	12,859	104,039	7

## Data Availability

All links to publicly archived datasets analyzed during the study are provided in “Materials and Methods” section. Additional data is provided in the Supplementary Materials. The analytical pipeline, examples, and jupyter notebooks are freely available at https://github.com/starchevskayamaria17/uncoVir, accessed on December 2022.

## Supplementary Materials

The following supporting information can be downloaded at: https://www.mdpi.com/article/10.3390/v15020395/s1, Figure S1: Distribution of contigs by non-viral families in genomic (top) and transcriptomic (bottom) samples. Total number of annotated contigs: 13,314; Figure S2: Distribution of contigs by non-viral classes in genomic (top) and transcriptomic (bottom) samples. Total number of annotated contigs: 13,314; Figure S3: Gel electrophoresis of PCR products showing the presence of the first bracoviral fragment in Colorado potato beetle tissues (primer pairs 1-4). Corresponding bracoviral fragment and primer sequences are shown in Supplementary Table S2; Figure S4: Gel electrophoresis: PCR products showing the presence of the second bracoviral fragment in Colorado potato beetle tissues (primer pairs 11-15). Corresponding bracoviral fragment and primer sequences are shown in Supplementary Table S2; Figure S5: Gel electrophoresis: PCR products showing no insertion of the first bracoviral fragment into the Colorado potato beetle or bracovirus genome (primer pairs 5-10). Corresponding bracoviral fragment and primer sequences are shown in Supplementary Table S2; Figure S6: Gel electrophoresis: PCR products showing no insertion of the second bracoviral fragment into the Colorado potato beetle or bracovirus genome (primer pairs 16-18). Corresponding bracoviral fragment and primer sequences are shown in Supplementary Table S2. Table S1: The summary of bioprojects used in this research (NCBI SRA); Table S2: Bracoviral genetic sequences and primers used to confirm the presence of certain viral fragments and inserts in the genome of Leptinotarsa decemlineata; Table S3: Contigs annotations with BLASTx (e-value < 10**-3, matches with bacteriophage proteins were excluded ); Table S4: Annotation BLASTx of virus-positive contigs with a known family (minimum e-value, maximum bitscore); Table S5: Annotation BLASTx of virus-positive contigs with a unknown family (minimum e-value, maximum bitscore); Table S6: Annotation BLASTx of virus-negative contigs (minimum e-value, maximum bitscore); Table S7: The summary of ST4 and ST5 tables. The distribution of virus-positive contigs by viral taxonomic groups; Table S8: Clusterization (Heat map); Table S9: Read alignment metrics for viral genomes in the BigViralDB database.
