# Supplementary material for: The Metagenomic Analysis of Viral Diversity in Colorado Potato Beetle Public NGS Data
Source: Viruses. 2023 Jan 30;15(2):395. doi: 10.3390/v15020395 (PMC9963324; doi:10.3390/v15020395)
Supplement: Supplementary file 1 [file viruses-15-00395-s001.zip › supplementary figures.pdf]

Supplementary Figures

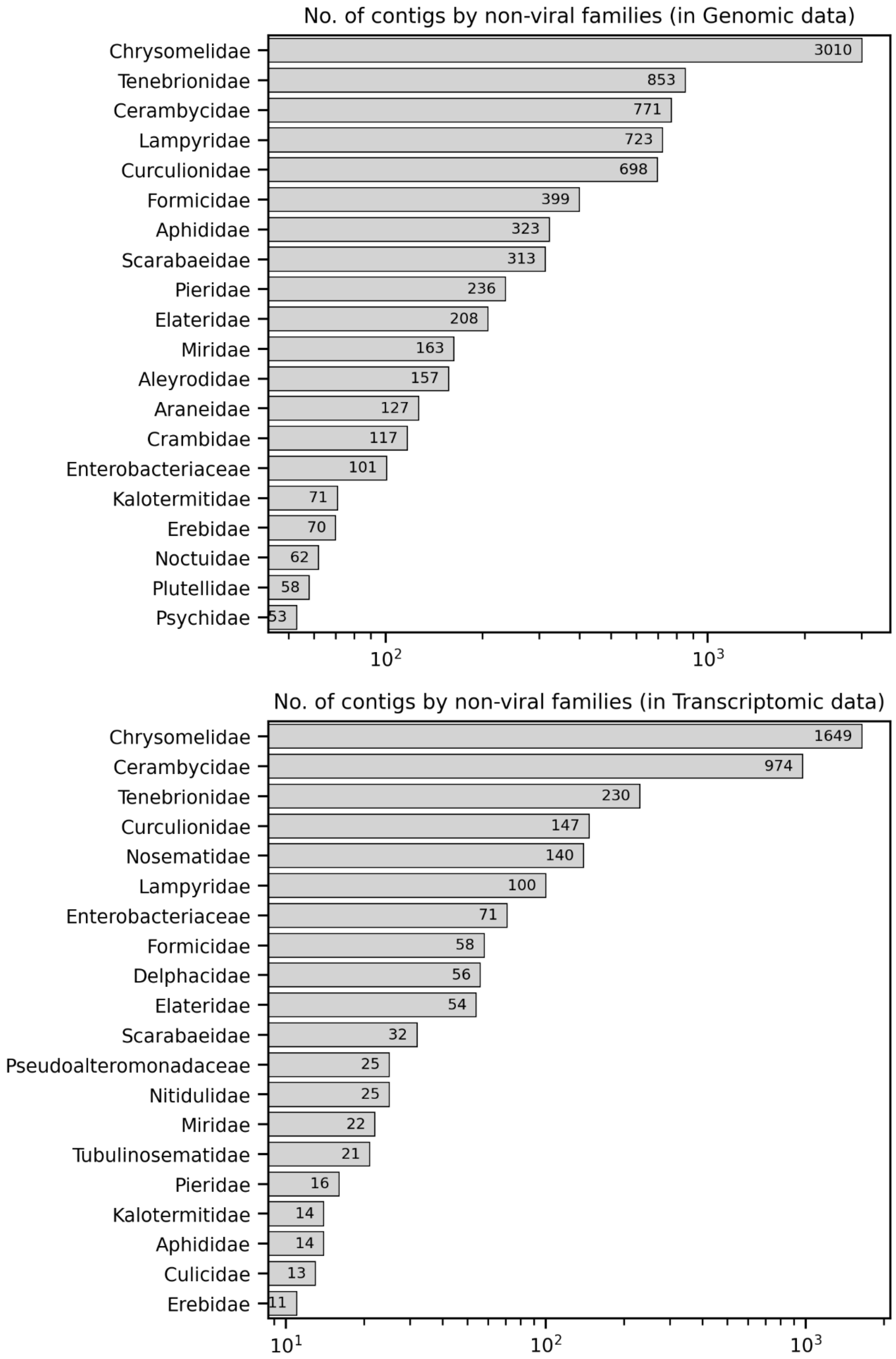

Figure S1. Distribution of contigs by non-viral families in genomic (top) and transcriptomic (bottom) samples. Total number of annotated contigs: 13,314.

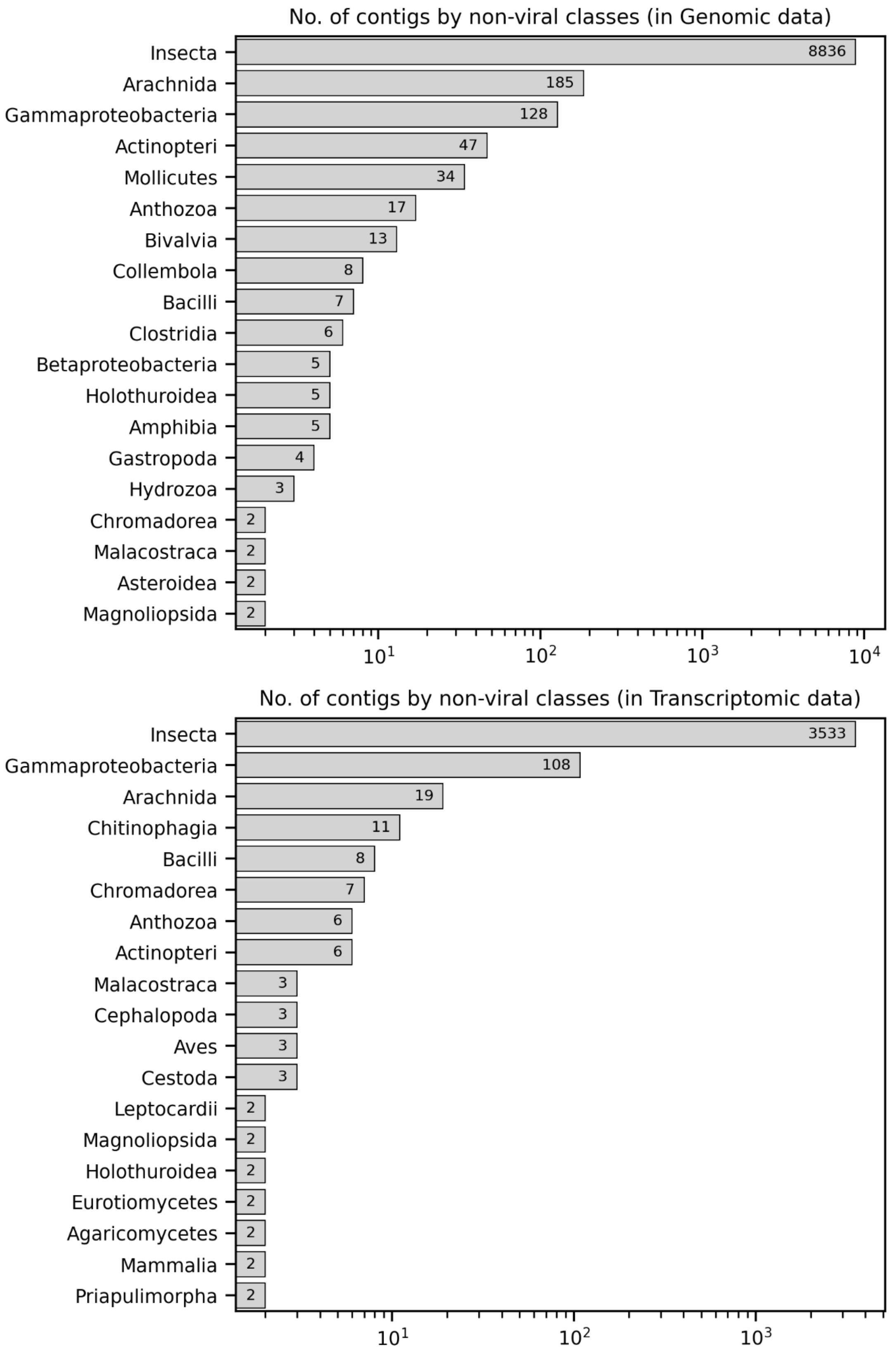

Figure S2. Distribution of contigs by non-viral classes in genomic (top) and transcriptomic (bottom) samples. Total number of annotated contigs: 13,314.

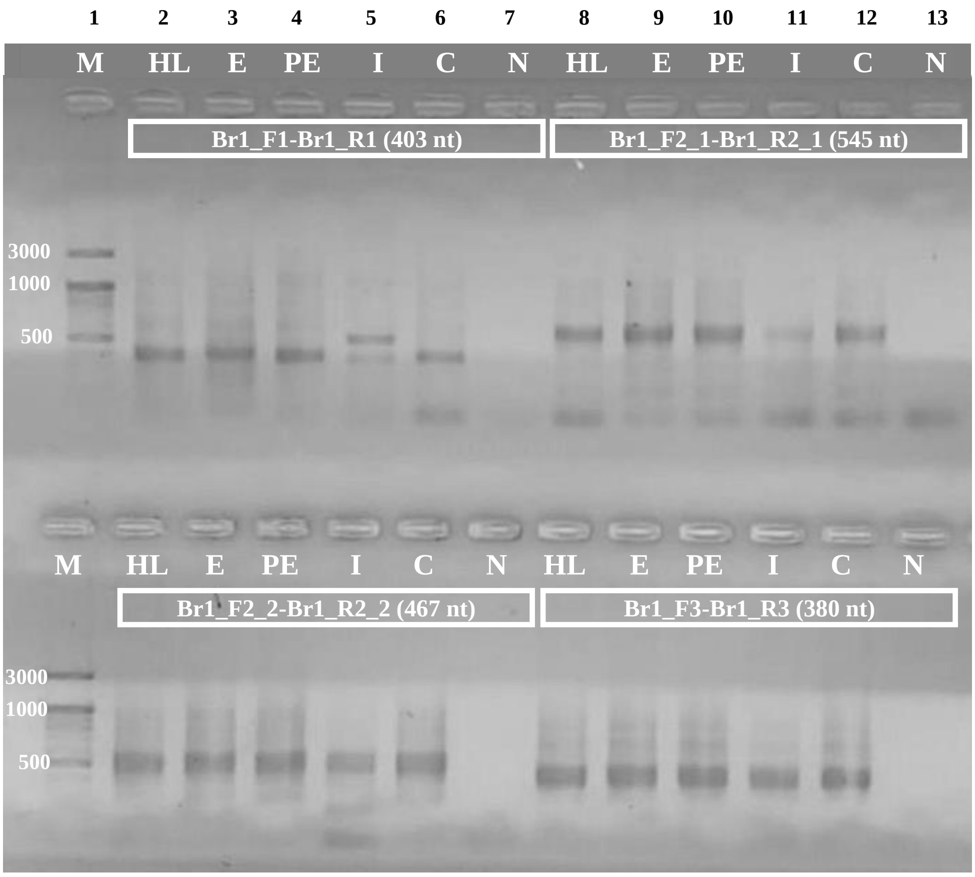

Figure S3. Gel electrophoresis of PCR products showing the presence of the first bracoviral fragment in Colorado potato beetle tissues (primer pairs 1-4). Corresponding bracoviral fragment and primer sequences are shown in Supplementary Table 2. In upper gel lane 1 contains marker (DNA ladder 1 kb, #M11, SibEnzyme, Russia), lanes 2-6 contain PCR products obtained with primers Br1_F1/Br1_R1 from different tissue samples (HL – hemolymp, E – eggs, PE – pure eggs, I – imago, C – cuticle from larva), lane 7 contains negative control sample, lanes 8-12 contain PCR products obtained with primers Br1_F2_1/Br1_R2_1 from different tissue samples (HL – hemolymp, E – eggs, PE – pure eggs, I – imago, C – cuticle from larva), lane 13 contains negative control sample. In lower gel lane 1 contains the same marker, lanes 2-6 contain PCR products obtained with primers Br1_F2_2/Br1_R2_2 from different tissue samples (HL – hemolymp, E – eggs, PE – pure eggs, I – imago, C – cuticle from larva), lane 7 contains negative control sample, lanes 8-12 contain PCR products obtained with primers Br1_F3/Br1_R3 from different tissue samples (HL – hemolymp, E – eggs, PE – pure eggs, I – imago, C – cuticle from larva), lane 13 contains negative control sample.

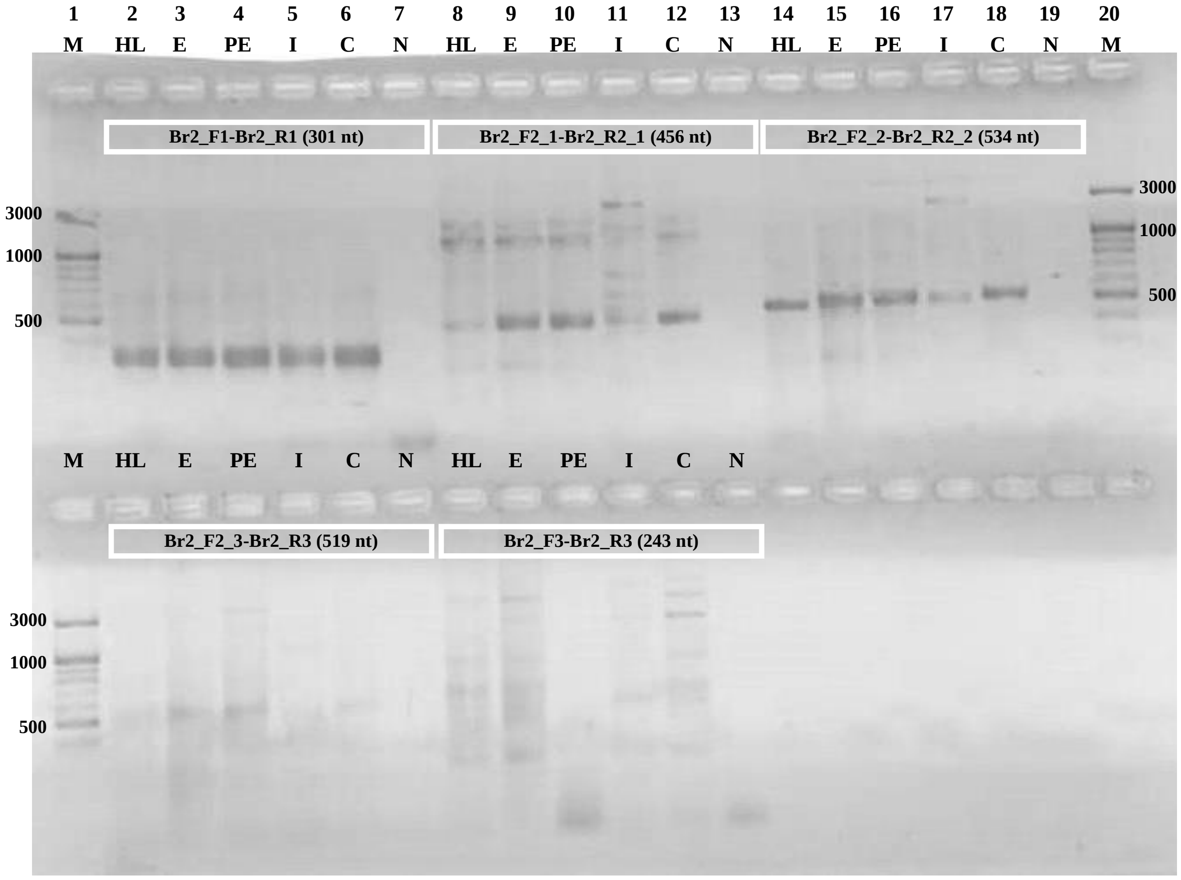

Figure S4. Gel electrophoresis: PCR products showing the presence of the second bracoviral fragment in Colorado potato beetle tissues (primer pairs 11-15). Corresponding bracoviral fragment and primer sequences are shown in Supplementary Table 2. In upper gel lane 1 contains marker (DNA ladder 1 kb, #M11, SibEnzyme, Russia), lanes 2-6 contain PCR products obtained with primers Br2_F1/Br2_R1 from different tissue samples (HL – hemolymp, E – eggs, PE – pure eggs, I – imago, C – cuticle from larva), lane 7 contains negative control sample, lanes 8-12 contain PCR products obtained with primers Br2_F2_1/Br2_R2_1 from different tissue samples (HL – hemolymp, E – eggs, PE – pure eggs, I – imago, C – cuticle from larva), lane 13 contains negative control sample, lanes 14-18 contain PCR products obtained with primers Br2_F2_2/Br2_R2_2 from different tissue samples (HL – hemolymp, E – eggs, PE – pure eggs, I – imago, C – cuticle from larva), lane 19 contains negative control, and lane 20 contains the marker. In lower gel lane 1 contains the same marker, lanes 2-6 contain PCR products obtained with primers Br2_F2_3/Br2_R3 from different tissue samples (HL – hemolymp, E – eggs, PE – pure eggs, I – imago, C – cuticle from larva), lane 7 contains negative control sample, lanes 8-12 contain PCR products obtained with primers Br2_F3/Br2_R3 from different tissue samples (HL – hemolymp, E – eggs, PE – pure eggs, I – imago, C – cuticle from larva), lane 13 contains negative control sample.

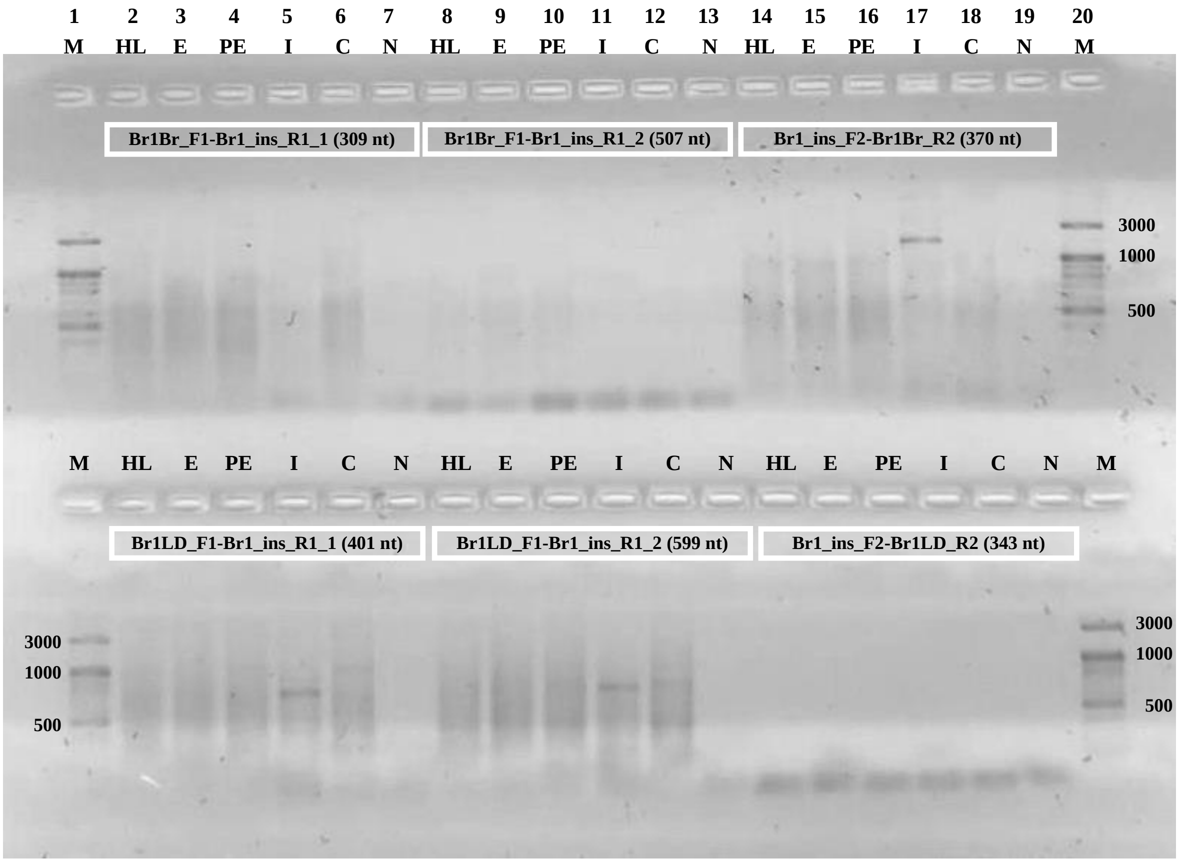

Figure S5. Gel electrophoresis: PCR products showing no insertion of the first bracoviral fragment into the Colorado potato beetle or bracovirus genome (primer pairs 5-10). Corresponding bracoviral fragment and primer sequences are shown in Supplementary Table 2. In upper gel lane 1 contains marker (DNA ladder 1 kb, #M11, SibEnzyme, Russia), lanes 2-6 contain PCR products obtained with primers Br1Br_F1/Br1_ins_R1_1 from different tissue samples (HL – hemolymp, E – eggs, PE – pure eggs, I – imago, C – cuticle from larva), lane 7 contains negative control sample, lanes 8-12 contain PCR products obtained with primers Br1Br_F1/Br1_ins_R1_2 from different tissue samples (HL – hemolymp, E – eggs, PE – pure eggs, I – imago, C – cuticle from larva), lane 13 contains negative control sample, lanes 14-18 contain PCR products obtained with primers Br1_ins_F2/Br1Br_R2 from different tissue samples (HL – hemolymp, E – eggs, PE – pure eggs, I – imago, C – cuticle from larva), lane 19 contains negative control, and lane 20 contains the marker. In lower gel lane 1 contains the same marker, lanes 2-6 contain PCR products obtained with primers Br1LD_F1/Br1_ins_R1_1 from different tissue samples (HL – hemolymp, E – eggs, PE – pure eggs, I – imago, C – cuticle from larva), lane 7 contains negative control sample, lanes 8-12 contain PCR products obtained with primers Br1LD_F1/Br1_ins_R1_2 from different tissue samples (HL – hemolymp, E – eggs, PE – pure eggs, I – imago, C – cuticle from larva), lane 13 contains negative control sample, lanes 14-18 contain PCR products obtained with primers Br1_ins_F2/Br1LD_R2 from different tissue samples (HL – hemolymp, E – eggs, PE – pure eggs, I – imago, C – cuticle from larva), lane 19 contains negative control, and lane 20 contains the marker.

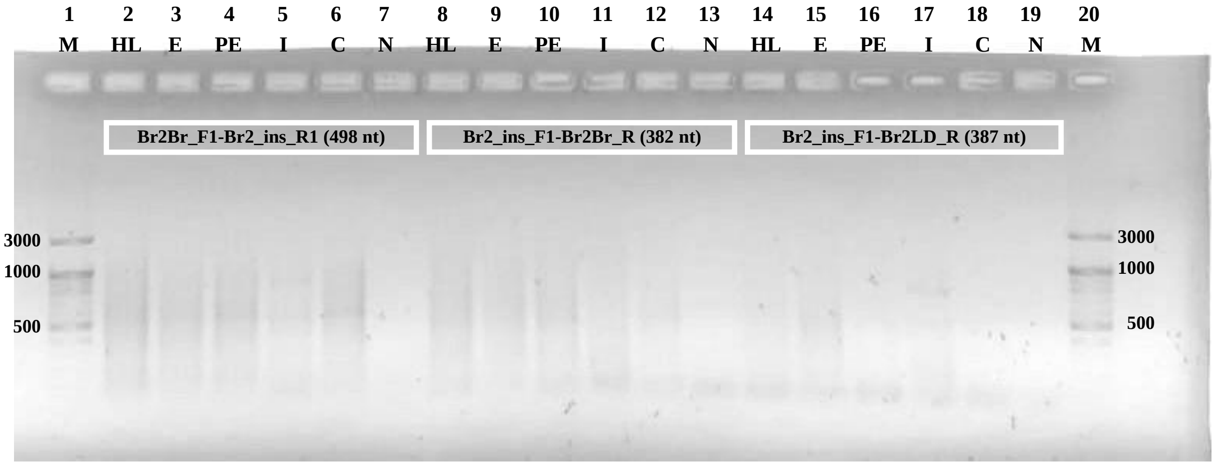

Figure S6. Gel electrophoresis: PCR products showing no insertion of the second bracoviral fragment into the Colorado potato beetle or bracovirus genome (primer pairs 16-18). Corresponding bracoviral fragment and primer sequences are shown in Supplementary Table 2. Lane 1 contains marker (DNA ladder 1 kb, #M11, SibEnzyme, Russia), lanes 2-6 contain PCR products obtained with primers Br2Br_F1/Br2_ins_R1 from different tissue samples (HL – hemolymp, E – eggs, PE – pure eggs, I – imago, C – cuticle from larva), lane 7 contains negative control sample, lanes 8-12 contain PCR products obtained with primers Br2_ins_F1/Br2Br_R from different tissue samples (HL – hemolymp, E – eggs, PE – pure eggs, I – imago, C – cuticle from larva), lane 13 contains negative control sample, lanes 14-18 contain PCR products obtained with primers Br2_ins_F1/Br2LD_R from different tissue samples (HL – hemolymp, E – eggs, PE – pure eggs, I – imago, C – cuticle from larva), lane 19 contains negative control, and lane 20 contains the marker.
